# Screening for HIV Among Patients at Tuberculosis Clinics — Results from Population-Based HIV Impact Assessment Surveys, Malawi, Zambia, and Zimbabwe, 2015–2016

**DOI:** 10.15585/mmwr.mm7010a2

**Published:** 2021-03-12

**Authors:** Nikhil Kothegal, Alice Wang, Sasi Jonnalagadda, Adam MacNeil, Elizabeth Radin, Kristin Brown, Owen Mugurungi, Regis Choto, Shirish Balachandra, John H. Rogers, Godfrey Musuka, Thokozani Kalua, Michael Odo, Andrew Auld, Laurence Gunde, Evelyn Kim, Danielle Payne, Patrick Lungu, Lloyd Mulenga, Ahmed Saadani Hassani, Tepa Nkumbula, Hetal Patel, Bharat Parekh, Andrew C. Voetsch

**Affiliations:** ^1^Public Health Institute, Oakland, California; ^2^Division of Global HIV and TB, Center for Global Health, CDC; ^3^ICAP at Columbia University, New York; ^4^Zimbabwe Ministry of Health; ^5^Division of Global HIV and TB, Center for Global Health, CDC, Zimbabwe; ^6^ICAP at Columbia University, Zimbabwe; ^7^Department of HIV/AIDS and Viral Hepatitis, Malawi Ministry of Health; ^8^Division of Global HIV and TB, Center for Global Health, CDC, Malawi; ^9^National Tuberculosis Programme, Zambia Ministry of Health; ^10^Division of Global HIV and TB, Center for Global Health, CDC, Zambia; ^11^ICAP at Columbia University, Zambia.

The World Health Organization and national guidelines recommend HIV testing and counseling at tuberculosis (TB) clinics for all patients, regardless of TB diagnosis (*1*). Population-based HIV Impact Assessment (PHIA) survey data for 2015–2016 in Malawi, Zambia, and Zimbabwe were analyzed to assess HIV screening at TB clinics among persons who had positive HIV test results in the survey. The analysis was stratified by history of TB diagnosis* (presumptive versus confirmed^†^), awareness^§^ of HIV-positive status, antiretroviral therapy (ART)^¶^ status, and viral load suppression among HIV-positive adults, by history of TB clinic visit. The percentage of adults who reported having ever visited a TB clinic ranged from 4.7% to 9.7%. Among all TB clinic attendees, the percentage who reported that they had received HIV testing during a TB clinic visit ranged from 48.0% to 62.1% across the three countries. Among adults who received a positive HIV test result during PHIA and who did not receive a test for HIV at a previous TB clinic visit, 29.4% (Malawi), 21.9% (Zambia), and 16.2% (Zimbabwe) reported that they did not know their HIV status at the time of the TB clinic visit. These findings represent missed opportunities for HIV screening and linkage to HIV care. In all three countries, viral load suppression rates were significantly higher among those who reported ever visiting a TB clinic than among those who had not (p<0.001). National programs could strengthen HIV screening at TB clinics and leverage them as entry points into the HIV diagnosis and treatment cascade (i.e., testing, initiation of treatment, and viral load suppression).

PHIA surveys are nationally representative, cross-sectional, household-based, two-stage cluster sample surveys designed to measure HIV program impact (*2*). During PHIA surveys, consenting persons aged ≥15 years were asked about ever visiting a TB clinic, HIV testing during their TB clinic visit (i.e., received a test, did not receive a test because they knew their HIV-positive status, or did not receive a test and did not know their HIV status), and TB diagnosis notification by a clinician. Interview data were used to classify persons as having presumptive or confirmed cases of TB. After the interview, persons underwent HIV testing in the household using the national HIV rapid test algorithm (followed by the laboratory-based Geenius HIV-1/2 confirmatory assay). Viral load testing and ART detection were conducted in a laboratory using procedures described previously (*2*,*3*).** 

Survey data were weighted to account for differential selection probabilities, with adjustments for nonresponse and undercoverage of the population by age and sex in each country. Estimated percentages were weighted, and confidence intervals were calculated using jackknife replicate weights. Via chi-square tests, p values <0.05 were considered statistically significant. SAS survey procedures (version 9.4; SAS Institute) were used for all analyses. This activity was reviewed by CDC and was conducted consistent with applicable federal law and CDC policy^††^ and was reviewed by Columbia University and local ethics boards in each country. 

The number of participants in the PHIA survey was 19,652 in Malawi, 21,280 in Zambia, and 22,490 in Zimbabwe.^§§^ Among those who had visited a TB clinic, 42.9% in Malawi, 30.7% in Zambia, and 28.5% in Zimbabwe reported that they were not screened for HIV during their TB clinic visit and did not know their HIV status. Among TB clinic attendees, an additional 9.1% in Malawi, 8.4% in Zambia, and 9.4% in Zimbabwe reported that they did not receive a test for HIV at the TB clinic because they already knew that they were HIV-positive (Table 1).

**TABLE 1:** Patients who had ever visited a tuberculosis (TB) clinic and ever received a TB diagnosis, by HIV status — Population-based HIV Impact Assessment (PHIA) surveys, Malawi, Zambia, and Zimbabwe, 2015–2016

Characteristic	Weighted % (95% CI)		
	Malawi (n = 19,652)	Zambia (n = 21,280)	Zimbabwe (n = 22,490)
**Ever visited a TB clinic**			
**All**	**4.7 (4.3–5.1)**	**6.7 (6.3–7.1)**	**9.7 (9.2–10.3)**
HIV-positive*	18.4 (16.4–20.4)	23.5 (21.4–25.6)	32.6 (30.6–34.6)
HIV-negative*	3.0 (2.6–3.3)	4.3 (3.9–4.6)	5.9 (5.5–6.4)
Never received testing for HIV	4.8 (3.8–5.8)	7.3 (5.9–8.7)	8.4 (7.0–9.9)
**Received testing for HIV at TB clinic**			
Yes	48.0 (44.3-51.7)	60.9 (57.9–63.9)	62.1 (59.7–64.5)
No			
Did not know HIV status at TB clinic visit	42.9 (39.1-46.8)	30.7 (27.9–33.5)	28.5 (26.3–30.7)
Knew HIV-positive status during TB clinic visit	9.1 (7.1–11.0)	8.4 (6.9–10.0)	9.4 (8.0–10.8)
**Ever received a TB diagnosis**			
**All**	**1.6 (1.4–1.8)**	**2.5 (2.2–2.7)**	**3.2 (2.9–3.5)**
HIV-positive*	8.7 (7.3–10.1)	12.8 (11.2–14.4)	15.1 (13.7–16.4)
HIV-negative*	0.9 (0.6–1.1)	1.2 (1.0–1.4)	1.3 (1.1–1.6)
Never received testing for HIV	1.0 (0.6–1.5)	1.1 (0.5–1.7)	1.8 (1.2–2.4)

**Abbreviation:** CI = confidence interval.

* HIV status as determined by PHIA survey HIV confirmatory testing.

Among participants who received positive HIV test results during PHIA and who also reported visiting a TB clinic, 47.7% (Malawi) to 64.4% (Zimbabwe) reported receiving? HIV testing at the TB clinic (Table 2). Among participants who received positive HIV test results during PHIA and who did not receive a test for HIV at a previous TB clinic visit, 29.4% (Malawi), 21.9% (Zambia), and 16.2% (Zimbabwe) reported that they did not know their HIV status at the time of the TB clinic visit. These weighted percentages extrapolate to 47,383 HIV-positive persons in Malawi, 48,693 in Zambia, and 59,481 in Zimbabwe (Table 2) and represent an upper limit of HIV-positive persons in each country who might have been HIV-positive but were not screened during their TB clinic visit and remained without a diagnosis until the PHIA survey.

**TABLE 2:** Percentage of HIV-positive survey participants with previous TB clinic visit who reported that they did not receive HIV testing at that clinic visit — Population-based HIV Impact Assessment (PHIA) surveys, Malawi, Zambia, and Zimbabwe, 2015–2016

Characteristic	Malawi (n = 456)		Zambia (n = 580)		Zimbabwe (n = 1,071)	
	Weighted % (95% CI)	Weighted frequency (95% CI)^§^	Weighted % (95% CI)	Weighted frequency (95% CI)^§^	Weighted % (95% CI)	Weighted frequency (95% CI)^§^
**Received HIV testing at TB clinic**	47.7 (41.9–53.4)	76,835 (63,834-89,835)	58.1 (53.0–63.1)	128,811 (109,454–148,168)	64.4 (60.7–68.0)	236,904 (211,028–262,780)
**Did not receive HIV testing at TB clinic**	52.3 (46.6–58.1)	84,410 (70,202–98,619)	41.9 (36.9–47.0)	93,068 (79,070–107,066)	35.6 (32.0–39.3)	131,219 (115,021–147,417)
Known HIV-positive status*	23.0 (18.2–27.7)	37,027 (27,939–46,115)	20.0 (16.3–23.7)	44,375 (34,987–53,763)	19.5 (16.6–22.3)	71,738 (60,235–83,241)
Unknown HIV status*	29.4 (24.1–34.6)	47,383 (37,126–57,640)	21.9 (18.2–25.7)	48,693 (39,530–57,856)	16.2 (13.5–18.8)	59,481 (48,788–70,174)
Aware of HIV status during PHIA^†^	89.4 (81.2–97.7)	42,375 (32,655–52,096)	78.8 (70.7–87.0)	38,389 (29,965–46,812)	81.5 (74.5–88.4)	48,456 (39,330–57,582)
Unaware of HIV status during PHIA^†^	10.6 (2.3–18.8)	5,008 (798.2–9,218)	20.4 (12.3–28.5)	9,926 (5,665–14,186)	18.5 (11.6–25.5)	11,025 (6,209–15,481)

**Abbreviation:** CI = confidence interval.

* The number of persons with known HIV-positive status and those with unknown HIV status add up to the number that did not receive HIV testing at a TB clinic.

^†^ Persons who were aware of their HIV status during PHIA and those who were unaware of their HIV status during PHIA are among those with unknown HIV status during the TB clinic visit.

^§^ The weighted frequency was estimated by using survey weights based on age and sex distribution of the national population for each country.

Among participants who received positive HIV test results during PHIA and who reported not receiving an HIV test and not knowing their HIV status at the TB clinic visit, 10.6% (Malawi), 20.4% (Zambia), and 18.5% (Zimbabwe) were unaware of their HIV-positive status before the PHIA survey. These percentages correspond to 5,008 of 47,383 in Malawi, 9,926 of 48,693 in Zambia, and 11,025 of 59,481 in Zimbabwe (Table 2). In all three countries, viral load suppression rates were higher among TB clinic attendees with a confirmed TB diagnosis (Malawi, 87.1%; Zambia, 76.1%; Zimbabwe, 72.9%) and TB clinic attendees with presumptive TB (Malawi, 77.3%; Zambia, 74.0%; Zimbabwe, 72.7%) than among those who never visited a TB clinic (Malawi, 60.2%; Zambia, 49.6%; Zimbabwe, 50.8%) (Figure).

**FIGURE:**
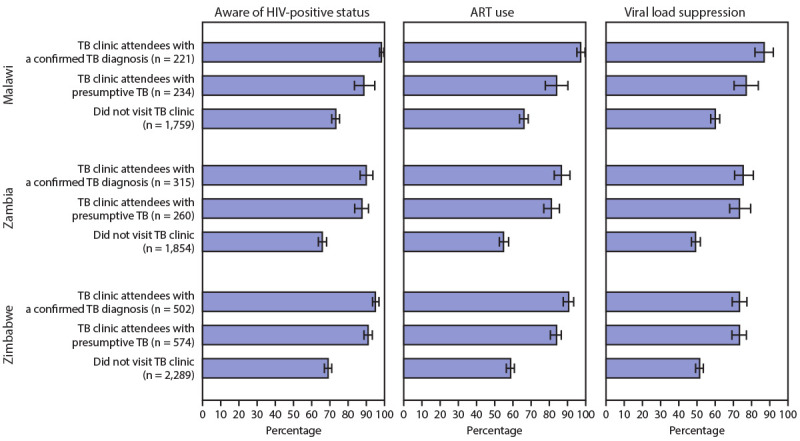
Awareness of HIV-positive status, antiretroviral therapy (ART) use, and viral load suppression, by tuberculosis (TB) clinic visit and TB diagnosis status **—** Population-based HIV Impact Assessment surveys, Malawi,* Zambia,^†^ and Zimbabwe,^§^ 2015–2016^¶^ * In Malawi, awareness of HIV-positive status and ART use were significantly different among those with and without a TB diagnosis (p<0.001 for both). † In Zambia, awareness of HIV-positive status and ART use were not significantly different among those with and without a TB diagnosis (p = 0.35 and p = 0.15, respectively). ^§^ In Zimbabwe, awareness of HIV-positive status and ART use were significantly different among those with and without a TB diagnosis (p = 0.01 for both). ^¶^ Confidence intervals shown by error bars.

In Malawi and Zimbabwe, awareness of HIV-positive status was significantly higher (p<0.001 and p = 0.01, respectively) among TB clinic attendees with a confirmed TB diagnosis (98.7% and 95.3%, respectively) than among those with presumptive TB (89.0% and 91.1%, respectively). Similarly, in Malawi and Zimbabwe, ART use was significantly higher (p<0.001 and p = 0.01, respectively) among TB clinic attendees with a confirmed TB diagnosis (97.0% and 90.8%, respectively) than among those with presumptive TB (83.8% and 83.9%, respectively). In Zambia, awareness of HIV-positive status or ART use did not significantly differ by TB diagnosis (Figure).

## Discussion

Across these three countries, the percentage of TB clinic attendees who reported having been screened for HIV during a TB clinic visit ranged from 48.0% to 62.1%, highlighting a gap in screening, despite the World Health Organization and national recommendations for universal HIV testing at TB clinics (*1*). Among the TB clinic attendees who received positive HIV test results during PHIA, a similar proportion self-reported having been screened for HIV at a TB clinic visit (47.7%–64.4%) (Table 2). Previous studies found that provider-initiated HIV testing and counseling among patients with presumptive TB are feasible and acceptable and are associated with increased HIV testing and improved identification of HIV-positive patients (*4*–*7*). In addition, HIV testing and linkage to care have been shown to improve TB treatment outcomes (*8*,*9*).

Results from the three PHIA surveys identified substantial gaps in HIV screening at TB clinics, with 16.2%–29.4% of participants who received positive HIV test results during PHIA reporting that they were not screened for HIV and did not know their HIV status at the time of their TB clinic visit (Table 2). Of these, 10.6%–20.4% were unaware of their HIV-positive status at the time of the PHIA survey.

HIV-positive adults who reported having visited a TB clinic also had significantly higher levels of awareness of their HIV status, ART use, and viral load suppression than did those who never visited a TB clinic. HIV-positive patients with TB might be more likely to seek TB care than are HIV-negative patients with TB. However, an analysis using cross-sectional survey data from Kenya postulated that TB might serve as an indicator disease leading to HIV diagnosis and ART initiation (*10*). This analysis also found that ART coverage was higher among HIV-positive adults with a confirmed TB diagnosis than among those without a previous diagnosis of TB. The PHIA data in Malawi and Zimbabwe also showed that awareness of HIV status and ART use were higher among those with a diagnosis of TB than among presumptive TB patients. These studies suggest that TB clinics, like antenatal care services, might serve as entry points to facilitate HIV diagnosis and care.

The findings in this report are subject to at least two limitations. First, the PHIA questionnaire did not include the TB clinic visit date or the reason for the TB clinic visit. Participants might have received an HIV diagnosis via HIV testing at a TB clinic or might have been referred to a TB clinic by their HIV care provider. Second, for those who received positive HIV test results during PHIA but reported not undergoing HIV screening at the TB clinic and not knowing their HIV status, HIV infection might have occurred before or after the TB clinic visit.

This analysis highlights coverage and gaps in HIV testing in TB clinics in three sub-Saharan African countries. The data suggest an association between HIV screening at TB clinics and improved clinical outcomes (awareness of HIV-positive status, ART use, and viral load suppression) for HIV-positive patients. Ensuring that all patients are screened for HIV at TB clinics can help identify HIV-positive persons and link them to care.

**Summary**

**What is already known about this topic?**

The World Health Organization recommends HIV testing and counseling at tuberculosis (TB) clinics for all patients, regardless of their TB diagnosis.

**What is added by the report?**

Population-based HIV Impact Assessment (PHIA) survey data from Malawi, Zambia, and Zimbabwe show that 16.2%–29.4% of HIV-positive persons were not screened for HIV during TB clinic visits; these visits represent missed opportunities for HIV diagnosis among persons who are not aware of their HIV-positive status.

**What are the implications for public health practice?**

HIV screening of patients with presumptive or confirmed TB could be strengthened to leverage TB clinics as entry points into the HIV care and treatment cascade.

## Footnotes

* Ascertained through self-report during the PHIA survey interview.

† Participants who reported visiting a TB clinic and receiving a TB diagnosis from a clinician were classified as having confirmed TB cases. Participants who reported visiting a TB clinic but did not receive a TB diagnosis from a clinician were classified as having presumptive cases.

§ Awareness of HIV-positive status was defined by self-report, with detectable ART, or both.

¶ Receiving ART was defined by self-reported ART use and/or detectable ART levels.

** Refers to the Malawi, Zimbabwe, and Zambia final reports.

†† 45 C.F.R. part 46, 21 C.F.R. part 56; 42 U.S.C. Sect. 241(d); 5 U.S.C. Sect. 552a; 44 U.S.C. Sect. 3501 et seq.

§§ Age range for eligible persons was 15–64 years in the Malawi and Zimbabwe PHIA surveys and 15–59 years in the Zambia PHIA survey.
